# The association between plasma IgG N-glycosylation and neonatal hypoxic–ischemic encephalopathy: a case-control study

**DOI:** 10.3389/fncel.2024.1335688

**Published:** 2024-03-20

**Authors:** Liangao Wang, Xinxia Lu, Meng Wang, Xuezhen Zhao, Peirui Li, Haitao Zhang, Qingtang Meng, Yujing Zhang, Yingjie Wang, Wei Wang, Long Ji, Haifeng Hou, Dong Li

**Affiliations:** ^1^School of Public Health, Shandong First Medical University and Shandong Academy of Medical Sciences, Jinan, China; ^2^Jinshan District Center for Disease Control and Prevention, Shanghai, China; ^3^Department of neonatology, Tai’an Maternal and Child Health Hospital, Tai’an, China; ^4^Department of obstetrics, Tai’an Maternal and Child Health Hospital, Tai’an, China; ^5^Centre for Precision Health, School of Medical and Health Sciences, Edith Cowan University, Perth, WA, Australia; ^6^College of Sports Medicine and Rehabilitation, Shandong First Medical University and Shandong Academy of Medical Sciences, Tai’an, China; ^7^The Second Affiliated Hospital of Shandong First Medical University, Tai'an, China

**Keywords:** hypoxic-ischemic encephalopathy, neonate, IgG, glycosylation, biomarker

## Abstract

**Introduction:**

Hypoxic-ischemic encephalopathy (HIE) is one of severe neonatal brain injuries, resulting from inflammation and the immune response after perinatal hypoxia and ischemia. IgG N-glycosylation plays a crucial role in various inflammatory diseases through mediating the balance between anti-inflammatory and pro-inflammatory responses. This study aimed to explore the effect of IgG N-glycosylation on the development of HIE.

**Methods:**

This case-control study included 53 HIE patients and 57 control neonates. An ultrahigh-performance liquid chromatography (UPLC) method was used to determine the features of the plasma IgG N-glycans, by which 24 initial glycan peaks (GPs) were quantified. Multivariate logistic regression was used to examine the association between initial glycans and HIE, by which the significant parameters were used to develop a diagnostic model. Though receiver operating characteristic (ROC) curves, area under the curve (AUC) and 95% confidence interval (CI) were calculated to assess the performance of the diagnostic model.

**Results:**

There were significant differences in 11 initial glycans between the patient and control groups. The levels of fucosylated and galactosylated glycans were significantly lower in HIE patients than in control individuals, while sialylated glycans were higher in HIE patients (*p* < 0.05). A prediction model was developed using three initial IgG N-glycans and fetal distress, low birth weight, and globulin. The ROC analysis showed that this model was able to discriminate between HIE patients and healthy individuals [AUC = 0.798, 95% CI: (0.716–0.880)].

**Discussion:**

IgG N-glycosylation may play a role in the pathogenesis of HIE. Plasma IgG N-glycans are potential noninvasive biomarkers for screening individuals at high risk of HIE.

## Introduction

Neonatal hypoxic–ischemic encephalopathy (HIE) is characterized by severe intrauterine hypoxia and abnormalities in cerebral blood flow, which can lead to extensive apoptosis of neonatal brain cells and ultimately to death (Torn et al., 2023). HIE is reported to put 15 per 10,000 live births at risk in high-income countries (Stoke et al., 2023).

HIE accounts for about 15–35% of all cases of brain damage in late-preterm neonates and disorders in late-preterm and term neonates and is associated with the potential for long-term complications, including epilepsy, motor impairment, and cognitive impairment (Wang et al., 2022). The pathological process of HIE includes three stages: early injury, intermediate stage, and reperfusion injury (Rasineni et al., 2022). A cascade of pathophysiological changes occurs during the reperfusion phase, and the inflammatory response induced by hypoxic stress plays a critical role in this process (Savman et al., 2013; Okazaki et al., 2023; Teo et al., 2023). Although hypothermic therapy is effective for neonatal HIE, the window of opportunity for treatment is narrow (Walas et al., 2020). Hypothermic therapy at 33–34°C for 72 h within 6 h of birth has been shown to be effective in reducing the risk of death or neurological disability (Mietzsch et al., 2020; Ovcjak et al., 2022). Therefore, clinicians must make a definitive diagnosis and take therapeutic measures within 6 h of birth. Currently, HIE is diagnosed by clinical manifestation criteria and neuroimaging using CT and MRI scans (Montaldo et al., 2019; Chen et al., 2022; Montaldo et al., 2023). However, the clinical manifestation criteria are a subjective approach to assessment. Neuroimaging is typically used in neonates between 3 and 10 days of life; therefore, to facilitate early diagnosis and targeted intervention, new biomarkers of neonatal HIE need to be explored.

Glycosylation is a type of posttranslational modification catalyzed by glycosyltransferases and glycosidases that affects the structure and function of proteins and lipids (Dall'Olio et al., 2013; Wang et al., 2019; Liu and Liu, 2021). The process of glycosylation involves the transfer of oligosaccharides to specific sites on proteins, resulting in the formation of glycoproteins (Shkunnikova et al., 2023). Immunoglobulin G (IgG), the most abundant immunoglobulin in human plasma and a major component of humoral immunity, plays a critical role in the inflammatory response of the human body. Studies have shown that IgG consists of a variable region [F(ab)_2_] and a fragmented crystalline region (Fc). There is one N-glycosylation site on the asparagine residue Asn297 in each heavy chain of the Fc region (Haslund-Gourley et al., 2023). This site serves to link different sugar residues, including galactose, sialic acid, core fucose, and bisecting GlcNAc (Hou et al., 2019). These glycan residues can activate antibody-dependent cell-mediated cytotoxicity (ADCC), complement-dependent cytotoxicity (CDC), and antibody-dependent cellular phagocytosis (ADCP), thereby affecting the functional properties of IgG (Quast et al., 2017).

While the N-glycosylation of IgG is relatively stable in healthy individuals, it undergoes changes in response to physiological or pathological changes (Gornik et al., 2009). Research has demonstrated that changes in IgG N-glycosylation are associated with several inflammation-related pathophysiological conditions and disease states, including autoimmune diseases, cardiovascular disease, and neurological disorders (Kronimus et al., 2019; Radovani et al., 2023; Wang et al., 2023). Identification of specific features of IgG N-glycosylation may provide a window of opportunity for early disease diagnosis (Liu et al., 2018). Therefore, we conducted this study to investigate changes in plasma IgG N-glycans associated with HIE and to assess the diagnostic performance of IgG N-glycosylation as potential biomarkers for HIE diagnosis.

## Materials and methods

### Participants

This case–control study included 53 HIE patients and 57 normal full-term neonates. All patients were recruited from the Tai’an Maternity and Child Health Hospital between October 2021 and November 2022.

The inclusion criteria for neonates were as follows: (1) full-term newborn and neonatal age ≤ 28 days. The exclusion criteria for neonates were as follows: (1) severe neonatal comorbidities, such as neonatal sepsis, central nervous system malformations and congenital heart disease; (2) maternal risk factors, such as alcohol, drugs and syphilis; and (3) incomplete data, including neonatal and maternal perinatal clinical data and blood samples used for experimental testing. Control infants with normal deliveries, normal Apgar scores, normal umbilical cord pH, and normal neonatal examination findings were recruited prenatally and concurrently. Biospecimens were collected prior to hypothermia treatment for the neonates in the case group. The Ethics Committee of Shandong First Medical University approved the study protocol.

### Diagnosis of hypoxic–ischemic encephalopathy

All the neonates with HIE included in our study met the diagnostic criteria established by the Chinese Pediatric Society Neonatology Group in 2005. Please refer to the Supplementary material for specific criteria.

### Demographic characteristics and clinical feature measurements

The characteristics of the study participants, including sex, gestational weeks, ethnicity, mode of delivery, and birth weight, were recorded by a doctor. Hematology and biochemical parameters, including aspartate aminotransferase (AST), globulin (GLO), total protein (TP), and albumin-globulin ratio (A/G), were determined with an automatic analyzer (Hitachi, Japan).

### Plasma IgG N-glycan analysis

Blood samples were collected using two tubes (2 mL) containing/without ethylene diamine tetra acetic acid (EDTA) by venipuncture after overnight fasting. Samples collected in EDTA-containing vacuum tubes were subjected to plasma separation for IgG N-glycans, while samples collected in EDTA-free tubes were subjected to serum separation for the determination of blood biochemical parameters.

The detailed experimental process and methods used for IgG N-glycan analysis were reported previously and followed established protocols in the field (Liu et al., 2023). The measurement of IgG N-glycans involved four steps: (1) IgG isolation; (2) N-glycan release and enrichment; (3) glycan labeling and cleanup; and (4) UPLC analysis. We have included the specific experimental steps in the Supplementary material.

After obtaining chromatograms for each sample, we divided the chromatograms into 24 initial glycan peaks based on a standard glycan peak map. We then used a normalization method, where the area of each peak was divided by the total area under the chromatogram, to obtain 24 glycan peak (GP) values for each sample. Additionally, the 24 Gp values were grouped into 17 derived traits using specific formulas (Qin et al., 2019). These derived traits were categorized into core fucosylation, galactosylation, sialylation, and bisecting GlcNAc. The formulas and categorization strategy for these derived traits are detailed in Supplementary Table S1.

### Statistical analysis

We used the Kolmogorov–Smirnov test to assess the normality of the data distribution. Continuous variables are presented as the mean ± standard deviation for normally distributed variables and as the median (*P*25–*P*75) for non-normally distributed variables. Categorical variables are expressed as n (%). To compare group differences for normally distributed data, we employed Student’s *t*-test. For skewed variables, we utilized the Wilcoxon rank-sum test. The chi-square test was applied to examine differences in categorical variables between groups.

We conducted a multivariable logistic regression analysis to determine the associations between HIE and the 24 initial glycans as well as the 17 derived features, adjusting for covariate effects such as fetal distress, low birth weight, and globulin. Considering that the inter-correlation between IgG N-glycosylation may lead to multicollinearity, we screened glycan biomarkers for the diagnosis of HIE patients in stepwise logistic regression analysis downscaling. A diagnostic model for diagnosing HIE patients was finally constructed. The diagnostic performance was evaluated by means of receiver operating characteristic (ROC) curve analysis, and the area under the curve (AUC) value, sensitivity, specificity, and Youden index were calculated. We used SPSS software version 25.0 (IBM, United States) and R software version 4.2.2 (R Core Team) for statistical analysis. GraphPad Prism (GraphPad Software, Inc., United States) and SPSS were used to plot boxplots and ROC curves, respectively. A two-tailed *p* value of < 0.05 was considered statistically significant.

## Results

### Participant characteristics

All included participants were Han Chinese, and all participants had complete demographic and N-glycan data. The demographic characteristics and biochemical indicators of the 53 HIE patients (30 males and 23 females) and 57 control individuals (31 males and 26 females) are listed in Table 1.

**Table 1:** Characteristics of the study participants.

Characteristics	HIE (*n* = 53)	Control (*n* = 57)	*t*/χ^2^	*p*
Age (days)	1.42 ± 2.22	1.58 ± 2.55	0.360	0.719
Male (male/female)	30/23	31/26	0.055	0.815
Gestational age (completed weeks, days)	39.14 ± 0.26	39.07 ± 0.34	1.411	0.598
Eutocia, *n* (%)	19 (35.8)	26 (45.6)	1.083	0.298
Premature rupture of membranes, *n* (%)	15 (28.3)	11 (19.3)	1.233	0.267
Fetal distress, *n* (%)	38 (71.7)	2 (3.5)	4.460	0.035*
Low birth weight, *n* (%)	20 (37.7)	9 (15.8)	6.814	0.009*
Respiratory distress, *n* (%)	23 (43.4)	21 (36.8)	0.492	0.483
AST (μ/L)	40.08 ± 15.567	46.32 ± 19.134	1.868	0.064
GLO (g/L)	14.99 ± 2.749	16.98 ± 3.534	3.273	0.001*
TP (g/L)	46.73 ± 5.278	49.66 ± 5.992	2.719	0.008*
A/G	2.19 ± 0.425	1.98 ± 0.336	−2.832	0.006*
Apgar score at 1 min	3 (2–5)	8 (7–10)		
Apgar score at 5 min	6 (3–7)	9 (8–10)		
Blood gas pH	6.92 (6.86–7.07)	7.28 (7.15–7.37)		

Data are shown as the mean ± standard deviation and n (proportion). *p* values were calculated by the independent-sample *t* test or chi-square test, and a *p* value < 0.05 was considered significant.

HIE, hypoxic–ischemic encephalopathy; AST, aspartate transaminase; GLO, globulin; TP, total protein; A/G, albumin-globulin ratio.

There was no significant difference in the sex of the patients between the group of HIE patients and the control group. Fetal distress, low birth weight (LBW), globulin (GLO), total protein (TP), and A/G significantly differed between the two groups. Among them, the HIE group exhibited significantly lower levels of GLO, and TP compared to the control group. Furthermore, the A/G was significantly higher in the HIE group than in the control group. Additionally, the prevalence of fetal distress and low birth weight was significantly higher in HIE patients than in control individuals.

### Immunoglobulin G N-glycome composition in participants

**Figure 1:**
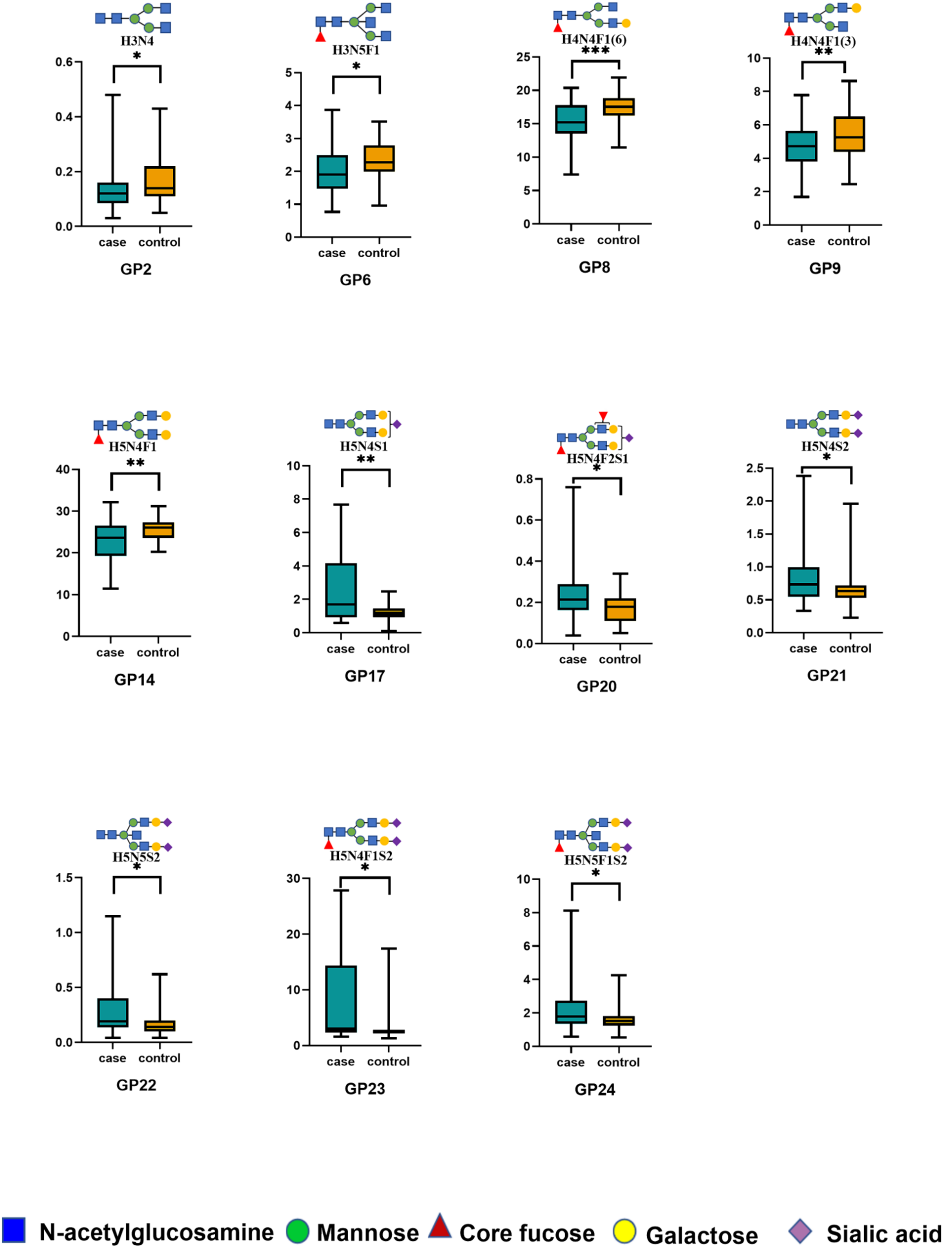
The relative abundance of 11 initial glycans with significant differences between the HIE and control groups. *p < 0.05; **p < 0.01; ***p < 0.001. *p* values were calculated with the Wilcoxon rank-sum test. Significant differences between HIE patients and control individuals in the directly measured glycan structures were observed. Data are shown as box plots. Each box represents the 25th to 75th percentile. The lines inside the box indicate the median. The lines outside the box indicate the 0th and 100th percentiles. GP, glycan peak; IgG, immunoglobulin G; HIE, hypoxic–ischemic encephalopathy.

Our UPLC assay initially detected 24 oligosaccharide chains that were bound to the IgG Fc domain. Normalized transformations were then applied to the data. Please refer to the Supplementary material for the distribution of the 24 initial glycans (Supplementary Table S2). Statistical analysis was undertaken employing the Wilcoxon rank-sum test to compare the initial glycan levels of the two groups. Of these, five initial glycans (GP2, GP6, GP8, GP9, and GP14) were significantly decreased in the HIE group compared with the control group (Figure 1), and six initial glycans (GP17, GP20, GP21, GP22, GP23, and GP24) were significantly increased in the HIE group compared with the control group (Figure 1).

In addition, to explore the potential role of IgG N-glycans in the development of HIE, we calculated 17 derived properties and categorized them into four major glycosylation features: fucosylation, galactosylation, sialylation, and bisecting N-acetylglucosamine. As presented in Supplementary Table S2, 10 of the 17 derived glycan characteristics differed between the HIE group and the control individuals. In terms of key glycosylation features, we observed a general decrease in fucosylation in HIE patients. Specifically, eight fucosylated glycans (GP6, GP8, GP9, GP14, F, FN, FG1, and FG2) showed a decrease, while two fucosylated glycans (GP23 and GP24) showed an increase. In the HIE group, there was a decrease in galactosylation (GP8, GP10, GP14, G1, and G2). Conversely, sialylation levels were increased (GP21, GP22, GP23, GP24, Stotal, S1, and S2). HIE patients also presented a significant reduction (GP6) and a significant increase (GP22 and GP24) in bisecting GlcNAc glycans. However, there was no significant difference in overall bisecting N-acetylglucosamine glycans between the HIE patients and the control group.

### Association of IgG N-glycans with hypoxic–ischemic encephalopathy

**Figure 2:**
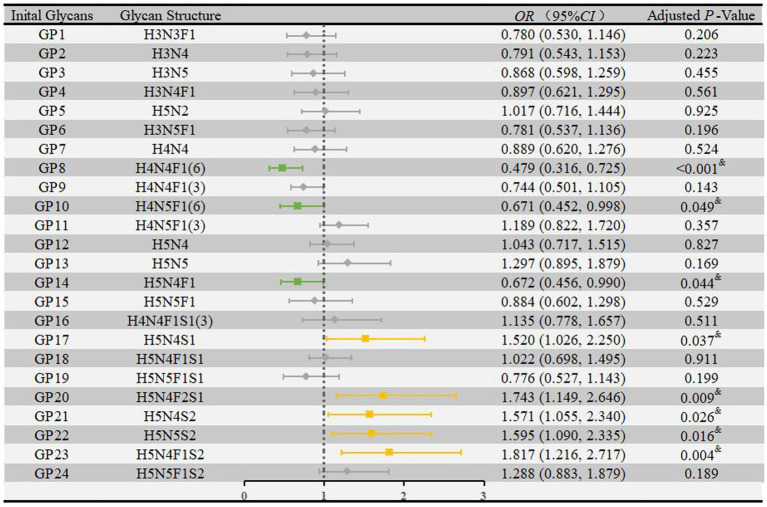
Associations of the normalized initial glycans and HIE as determined by multivariate logistic regression analyses. Multivariate logistic regression analyses were performed after adjusting for the effects of fetal distress, low birth weight, and globulin, and *p* < 0.05 was considered statistically significant using logistic regression analysis. HIE, hypoxic–ischemic encephalopathy; GP, glycan peak; OR, odds ratio; CI, confidence interval.

We performed multivariate logistic regression analyses to assess the associations of IgG N-glycans with HIE while adjusting for the effects of fetal distress, low birth weight, and globulin levels. As shown in Figure 2, eight glycans were significantly associated with HIE: GP8 [*OR* = 0.479, 95% *CI*: (0.316–0.725)], GP10 [*OR* = 0.671, 95% *CI*: (0.452–0.998)], and GP14 [*OR* = 0.672, 95% *CI*: (0.456–0.990)] were negatively correlated with HIE, and GP17 [*OR* = 1.520, 95% *CI*: (1.026–2.250)], GP20 [*OR* = 1.743, 95% *CI*: (1.149–2.646)], GP21 [*OR* = 1.571, 95% *CI*: (1.055–2.340)], GP22 [*OR* = 1.595, 95% *CI*: (1.090–2.335)], and GP23 [*OR* = 1.817, 95% *CI*: (1.216–2.717)] were positively correlated with HIE.

As shown in Supplementary Figure S1, seven derived traits were significantly associated with HIE: S_total_ [*OR* = 1.915, 95% *CI*: (1.270–2.889)] and S2 [*OR* = 1.699, 95% *CI*: (1.142–2.529)] were negatively correlated with HIE, while GPN [*OR* = 0.522, 95% *CI*: (0.346–0.788)], G1 [*OR* = 0.513, 95% *CI*: (0.340–0.774)], F [*OR* = 0.595, 95% *CI*: (0.395–0.896)], FG1 [*OR* = 0.511, 95% *CI*: (0.336–0.779)], and FG2 [*OR* = 0.672, 95% *CI*: (0.456–0.990)] were positively correlated with HIE.

### Identification of hypoxic–ischemic encephalopathy using IgG N-glycans and blood markers

**Figure 3:**
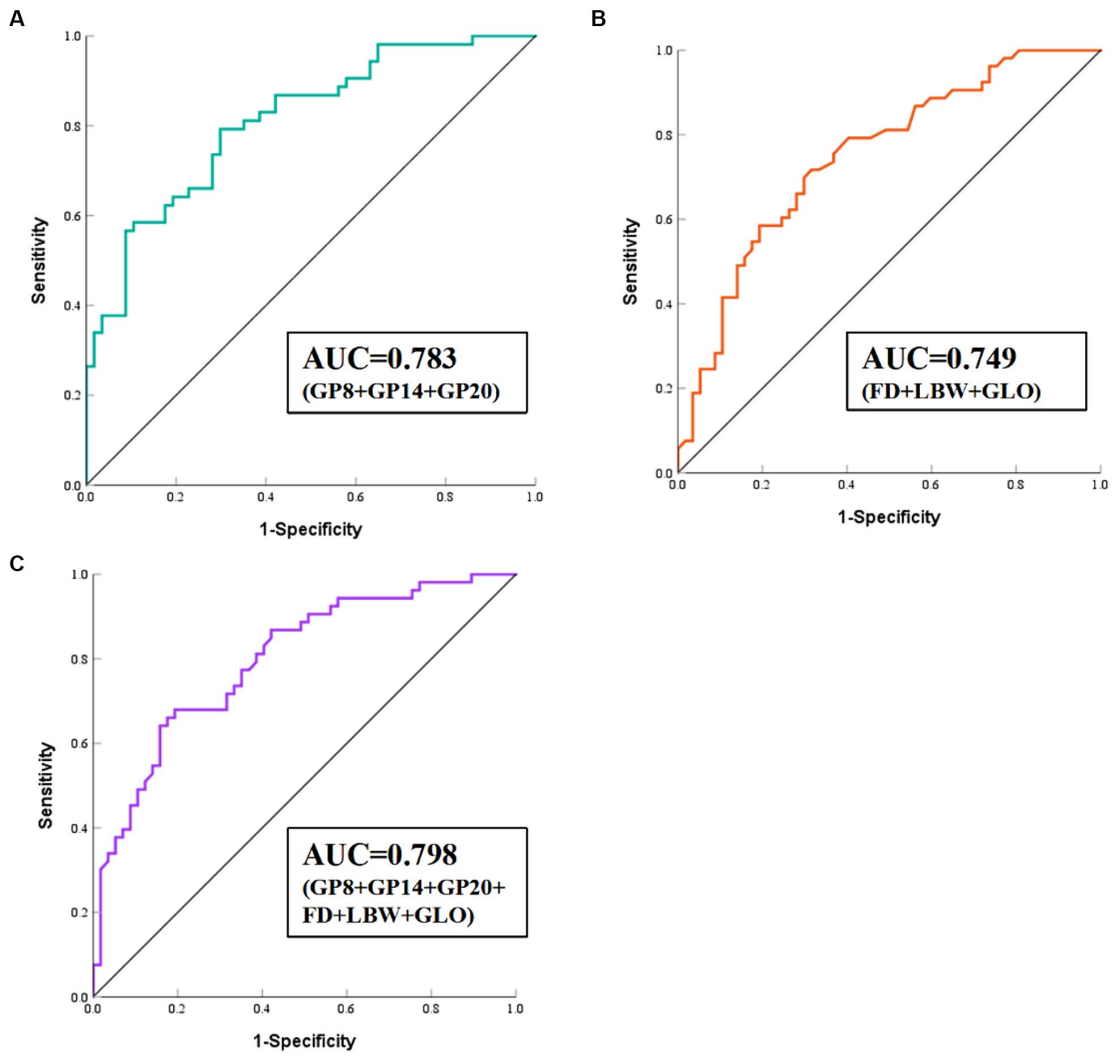
ROC curve analysis of the binary logistic regression model for the prediction of HIE. **(A)** Model 1 consists of GP8, GP14, and GP20. **(B)** Model 2 consists of FD, LBW, and GLO. **(C)** Model 3 consists of GP8, GP14, and GP20, FD, LBW, and GLO. HIE, Hypoxic–ischemic encephalopathy; ROC, receiver operating characteristic; FD, fetal distress; LBW, low birth weight; GLO, globulin.

We developed a diagnostic model to distinguish HIE cases from control individuals. Further internal correlation analysis between these eight glycans revealed significant correlation coefficients ranging from 0.22 to 0.85, suggesting internal relationships between these glycans that could influence the statistical model (Supplementary Figure S2). To reduce dimensionality, stepwise regression was performed to select significant glycans (Supplementary Table S3), resulting in 3 glycans (GP8, GP14, GP20) being included in the diagnostic model (Supplementary Table S4; Figure 3), yielding an *AUC* value of 0.783 (95% *CI*: 0.694–0.871). Similarly, we included the differential conventional risk factor indicators (fetal distress, low birth weight, and globulin) of the patients in the model (Supplementary Table S4; Figure 3). The resulting *AUC* value for the model was 0.749 (95% *CI*: 0.658–0.840). We also examined the combined predictive value of the three initial glycans and three differential conventional risk factor biomarkers (Supplementary Table S4; Figure 3). The combined *AUC* value was calculated to be 0.798 (95% *CI*: 0.716–0.880), indicating improved predictive performance when initial glycans and conventional risk factors were considered together.

## Discussion

Our study found that infants with HIE had lower levels of fucosylated and galactosylated IgG N-glycans and higher levels of sialylation than healthy infants. These changes might impact the inflammatory status of damage caused by hypoxia among HIE infants. We developed a diagnostic model for the early detection of HIE that incorporates initial glycans and conventional risk factors, which has the potential to overcome the limitations associated with delayed clinical neurological assessment.

Neuroinflammatory responses in the brain play a critical role in the pathogenesis of HIE and are involved in both early and late brain damage, as well as in the repair and recovery of brain tissue after hypoxic–ischemic injury. Previous studies have shown that ischemic and hypoxic insults activate various inflammatory responses in the brain (Zhou et al., 2022). Microglia, specialized immune cells in the central nervous system, are key mediators of neuroinflammation (Bernis et al., 2022). During injury, such as LPS-induced inflammation or hypoxic–ischemic events, M1-phenotype microglia are rapidly activated, proliferate, and migrate to the site of injury, where they release various cytokines, chemokines, reactive oxygen species, and excitotoxic molecules (Douglas-Escobar and Weiss, 2015). It was found that HIE patients had a significant increase in the expression of inflammatory cytokines (IL-1β and TNF-α), several times higher than control individuals, with IL-6 expression increased by 57-fold (Chalak et al., 2014). Similarly, in an *in vivo* study, elevated levels of IL-6 and TNF-α were observed in a model of HIE-induced neurologic injury (Rocha-Ferreira et al., 2017). Aberrant expression of IgG N-glycosylation can modulate the pro- and anti-inflammatory states of the body (Radovani and Gudelj, 2022). Therefore, IgG N-glycosylation may play an important role in the progression of HIE and its underlying mechanisms.

IgG N-glycosylation, regulated by glycosyltransferases and glycosidases, is involved in the pathological processes of inflammatory diseases (Lu et al., 2023). The proinflammatory state exhibited by IgG with abnormal modifications plays a role in many neurological disorders (Kronimus et al., 2019). In a sample of atherosclerotic patients, those with vascular cognitive impairment showed a relative decrease in galactosylation levels compared to control individuals, along with an increase in inflammatory mediators (Wang et al., 2022). Ischemic stroke patients also exhibited changes in IgG glycosylation patterns that shifted toward proinflammatory (Wang et al., 2023). All these findings strongly suggest a close association between IgG N-glycosylation abnormalities and neurological disorders through the modulation of inflammatory responses.

The most prominent IgG glycosylation feature associated with HIE was a decrease in fucosylation. Fucosylation is relatively stable in the human body and is minimally influenced by age and sex (Larsen et al., 2021). They constitute 93.72% of total IgG glycans, and changes in fucosylation have a fundamental impact on IgG function (Li et al., 2022). The mechanisms underlying the regulation of the inflammatory response by fucosylation are relatively well understood. Reduced levels of fucosylation allow the Fc portion of IgG to bind to Fcγ-RIIIA receptors on immune cells. Fcγ-RIIIA is the major activating receptor on NK cells and mediates activation signaling for ADCC, thereby promoting proinflammatory responses (Golay et al., 2022). Our results showed a reduction in fucosylation, which is consistent with the findings reported in neonatal epilepsies. Furthermore, in a study of dementia, patients with cognitive impairment also showed a significant decrease in fucosylation compared to healthy individuals (Zhang et al., 2021).

The decrease in IgG N-glycan galactosylation inhibits the binding affinity between IgG and complement C1q required for activation of the classical pathway of complement, thereby suppressing CDC and exerting the proinflammatory function of IgG (Meng et al., 2022). Galactosylation plays an important role in many diseases, and this alteration has been reported in inflammatory bowel disease (Trbojevic et al., 2015), vascular cognitive impairment (Wang et al., 2022), and Parkinson’s disease (Russell et al., 2017). Our results showed a decrease in galactosylation, which is consistent with a study on ischemic stroke that also found a decrease in galactosylation (Liu et al., 2018). Furthermore, increased agalactosylation enhances the affinity between FcγRIII and serum mannose-binding lectin, leading to enhanced CDC activity and increased anti-inflammatory activity via alternative pathways (Gu et al., 2023). However, we did not observe statistically significant differences in the agalactosylation trait among the HIE patients.

Research has demonstrated that the presence of fucosylation, when combined with sialylation, significantly reduces ADCC and inhibits antibody-mediated cell killing *in vivo* (Lin et al., 2015). Conversely, in the absence of fucosylation, sialylation does not negatively affect ADCC. Recent studies have also suggested that sialylation exerts its anti-inflammatory effects through CDC mediation (Quast et al., 2015). Moreover, acute immune responses result in an increase in sialylation (Hou et al., 2021). Furthermore, another study demonstrated that sialylation has a minimal effect on the ADCC pathway in the absence of fucosylation (Li et al., 2017), which is consistent with our findings. These glycosylation abnormalities in IgG are thought to induce proinflammatory properties, which may partially explain the persistent inflammatory state that accompanies the development of HIE following asphyxia.

Numerous plasma biomarkers have been investigated to monitor the clinical progression of HIE (Caramelo et al., 2023). Currently, the IgG N-glycan assay offers a new diagnostic approach for inflammatory and immune diseases. Hypoxic–ischemic encephalopathy, which is a neuroinflammatory response resulting from the stress of oxygen deprivation in neonates, has the potential to alter the glycosylation of IgG Fc. The induction of inflammation by aberrant glycosylation may provide intriguing insights into the underlying mechanisms of HIE pathogenesis. Nonetheless, confirming causation presents challenges, as the observed alterations might stem from the disease itself rather than precipitating it.

To our knowledge, this study is the first to investigate the relationship between glycosylation profiles of circulating IgG and HIE in Chinese Han newborns, providing new insights into the pathogenesis of neonatal hypoxic–ischemic brain injury. However, this study has several limitations that are common to hospital-based case–control studies. First, we cannot demonstrate the causal relationships between IgG N-glycosylation and HIE due to the potential bias related to the study design. Second, as the incidence of HIE is low, the sample size of this study is relatively small, which limits the generalizability of this study and the robustness of the conclusions. In addition, the current investigation constitutes a preliminary pilot study aimed at elucidating the potential correlation between N-glycans and hypoxic–ischemic encephalopathy. To establish a more conclusive understanding of the intricate connections between N-glycan structures and HIE, it is imperative that subsequent cohort studies or Mendelian randomization studies with larger sample sizes are undertaken.

## Conclusion

Our findings suggest that the decreased fucosylation and galactosylation and increased sialylation of IgG Fc segments play a role in the development of HIE. IgG N-glycans may serve as potential biomarkers to differentiate HIE in infants, thereby improving the ability to diagnose HIE.

## Data availability statement

The raw data supporting the conclusions of this article will be made available by the authors, without undue reservation.

## Ethics statement

The studies involving humans were approved by The Ethics Committee of Shandong First Medical University. The studies were conducted in accordance with the local legislation and institutional requirements. Written informed consent for participation in this study was provided by the participants’ legal guardians/next of kin.

## Author contributions

LW: Investigation, Methodology, Project administration, Writing – original draft. XL: Methodology, Writing – original draft, Software, Validation. MW: Data curation, Formal Analysis, Writing – original draft. XZ: Formal Analysis, Supervision, Validation, Writing – original draft. PL: Data curation, Software, Writing – original draft. HZ: Data curation, Resources, Writing – original draft. QM: Data curation, Investigation, Writing – original draft. YZ: Software, Visualization, Writing – original draft. YW: Software, Visualization, Writing – original draft. WW: Supervision, Writing – original draft. LJ: Project administration, Resources, Writing – review & editing. HH: Project administration, Resources, Writing – review & editing. DL: Conceptualization, Funding acquisition, Project administration, Resources, Writing – review & editing.
