# Supplementary material for: The association between plasma IgG N-glycosylation and neonatal hypoxic–ischemic encephalopathy: a case-control study
Source: Front Cell Neurosci. 2024 Mar 20;18:1335688. doi: 10.3389/fncel.2024.1335688 (PMC10987743; doi:10.3389/fncel.2024.1335688)
Supplement: Supplementary file 1 [file Data_Sheet_1.docx]

**Diagnosis of hypoxic-ischemic encephalopathy**

According to clinical manifestations criteria and the craniocerebral computed tomography diagnostic standard for neonatal HIE established by the Neonatal Group of the Chinese Pediatric Society (2005), patients with the following four criteria can be diagnosed: (1) a clear history of abnormal obstetric conditions leading to fetal distress and severe fetal distress (fetal heart rate < 100 beats/min lasting more than 5 min; and/or III degree contamination of amniotic fluid) or an obvious history of asphyxia during delivery; (2) severe asphyxia at birth, defined as an Apgar score ≤ 3 points at 1 minute and still ≤ 5 points at 5 minutes and/or umbilical arterial blood gas pH ≤ 7.00 at birth; (3) nervous system symptoms that occurred soon after birth and lasted for more than 24 hours, such as changes in consciousness (excessive excitement, lethargy, coma), changes in muscle tension (increase or decrease), abnormal original reflexes (sucking, embrace reflex decrease or disappear), convulsions and brain stem signs (change in respiratory rhythm, change in pupation, slow reaction to light or disappearance) and increased AF tension; and (4) seizures caused by electrolyte disturbance, intracranial hemorrhage, and birth trauma, as well as brain damage caused by intrauterine infection, inherited metabolic diseases and other congenital diseases.

**The measurement of IgG N-glycans**

(1)A 100 μL plasma sample was added to a 96-well protein G monolithic plate ((CIM®r-Protein G 0.2ml Monolithic 96-well Plate, BIA Separations, Slovenia) for IgG isolation. Isolated IgG samples were denaturized with 1 mL of 0.1 mol∙L^-1^ formic acid and immediately neutralized with 1 mol∙L^-1^ ammonium bicarbonate.(2) For N-glycan release, 4 µL of PNGase F enzyme was added to the IgG samples and incubated in a 37 °C water bath for 18 h.(3) The released N-glycans were labeled with 2-aminobenzamide (2-AB) and then transferred into an oven at 65 °C for 3 h.(4) The 2-AB labeled glycans were analyzed with hydrophilic interaction liquid chromatography using an ultra-performance liquid chromatography (HILIC-UPLC) instrument (Walters Corporation, USA), by which 24 IgG glycan peaks (GPs) were detected. The structures of these GPs were identified by reference to a dataset established by mass spectrometry. The level of each glycan was quantitated by the percentage of the area of the relevant chromatogram peak to the integrated area of all glycans. The level of IgG galactosylation (referred to as the Gal ratio) was calculated from the relative intensity of agalactosylated (G0) vs. monogalactosylated (G1) and digalactosylated (G2) fucosylated biantennary glycans according to the formula G0/ (G1 + G2 × 2), as we previously described.

Table S1 The calculation formula of derived traits

| **Derived traits** | **Description** | **Computational method** |
| --- | --- | --- |
| GPN | Proportion of neutral glycans in total IgG glycans | GP1+GP2+GP3+GP4+GP5+GP6+GP7+GP8+GP9+GP10+GP11+GP12+GP13+GP14+GP15 |
| Stotal | Proportion of sialylated glycans in total IgG glycans | GP16+GP17+GP18+GP19+GP21+GP22+GP23+GP24 |
| S1 | Proportion of monosialylated glycans in total IgG glycans | GP16+GP17+GP18+GP19 |
| S2 | Proportion of disialylated glycans in total IgG glycans | GP21+GP22+GP23+GP24 |
| F | Proportion of fucosylated glycans in total IgG glycans | GP1+ GP4+ GP6+ GP8+ GP9+ GP10+ GP11+ GP14+ GP15+ GP16+ GP18+ GP19+ GP23+ GP24 |
| FN | Proportion of fucosylated glycans in total neutral IgG glycans | (GP1+ GP4+ GP6+ GP8+ GP9+ GP10+ GP11+ GP14+ GP15)/GPN*100 |
| FS | Proportion of fucosylated glycans in total sialylated IgG glycans | (GP16+ GP18+ GP19+ GP23+ GP24)/Stotal*100 |
| B | Proportion of bisecting glycans in total IgG glycans | GP3+ GP6+ GP10+ GP11+ GP13+ GP15+ GP19+ GP22+ GP24 |
| BN | Proportion of bisecting glycans in neutral IgG glycans | (GP3+ GP6+ GP10+ GP11+ GP13)/GPN*100 |
| BS | Proportion of bisecting glycans in sialylated IgG glycans | (GP19+ GP22+ GP24)/Stotal*100 |
| FG0 | Proportion of fucosylated agalactosylated glycans in total IgG glycans | GP4 |
| FG1 | Proportion of fucosylated monogalactosylated glycans in total IgG glycans | GP8 + GP9 |
| FG2 | Proportion of fucosylated galactosylated glycans in total IgG glycans | GP14 |
| G0 | Proportion of agalactosylated glycans in total IgG glycans | GP1+ GP2+ GP3+ GP4+ GP6 |
| G1 | Proportion of monogalactosylated glycans in total IgG glycans | GP7+ GP8+ GP9+ GP10+ GP11 |
| G2 | Proportion of galactosylated glycans in total IgG glycans | GP12+ GP13+ GP14+ GP15 |
| aGal/Gal ratio | the relative intensities of agalactosylated (G0) vs monogalactosyl (G1) and digalactosyl (G2) N-glycans | G0/ (G1 + G2*2) *100 |

B, bisecting GlcNAc; F, core fucose; G, galactose; N, neutral glycans; S, sialic acid.

Table S2 Comparison of IgG N- initial glycans and derived traits levels

| Variables | HIE (n=53) | Control (n=57) | Z | *P* |
| --- | --- | --- | --- | --- |
|  | M (*P*25–*P*75) | M (*P*25–*P*75) |  |  |
| Inital glycans |  |  |  |  |
| GP1 | 0.03 (0.02–0.06) | 0.04 (0.03–0.07) | -1.294 | 0.196 |
| GP2 | 0.12 (0.09–0.17) | 0.13 (0.11–0.22) | -2.148 | 0.032* |
| GP3 | 0.06 (0.04–0.10) | 0.08 (0.05–0.13) | -1.466 | 0.143 |
| GP4 | 8.55 (5.71–10.27) | 8.70 (7.29–10.73) | -1.313 | 0.189 |
| GP5 | 0.13 (0.09–0.26) | 0.14 (0.09–0.19) | -0.698 | 0.485 |
| GP6 | 2.01 (1.51–2.55) | 2.22 (1.95–2.77) | -1.962 | 0.049* |
| GP7 | 0.16 (0.08–0.22) | 0.18 (0.10–0.29) | -1.61 | 0.107 |
| GP8 | 15.17 (13.78–17.71) | 17.56 (16.21–18.87) | -4.217 | <0.001* |
| GP9 | 4.72 (3.86–5.63) | 5.20 (4.39–6.46) | -2.181 | 0.029* |
| GP10 | 4.03 (2.93–4.56) | 4.09 (3.84–4.52) | -1.81 | 0.07 |
| GP11 | 0.34 (0.23–0.56) | 0.33 (0.21–0.44) | -0.922 | 0.357 |
| GP12 | 1.50 (1.03–2.26) | 1.61 (1.20–2.06) | -0.212 | 0.832 |
| GP13 | 0.32 (0.24–0.42) | 0.24 (0.19–0.49) | -1.709 | 0.088 |
| GP14 | 23.66 (19.79–26.39) | 26.07 (23.73–27.36) | -3.123 | 0.002* |
| GP15 | 2.18 (1.77–2.45) | 2.13 (1.87–2.36) | -0.024 | 0.981 |
| GP16 | 2.27 (1.92–2.62) | 2.23 (1.99–2.67) | -0.051 | 0.959 |
| GP17 | 1.70 (1.06–4.02) | 1.18 (0.93–1.48) | -3.015 | 0.003* |
| GP18 | 19.28 (16.78–22.78) | 19.34 (17.32–21.48) | -0.523 | 0.601 |
| GP19 | 1.48 (1.28–1.68) | 1.45 (1.30–1.73) | -0.018 | 0.986 |
| GP20 | 0.24 (0.17–0.34) | 0.18 (0.11–0.22) | -3.796 | <0.001* |
| GP21 | 0.73 (0.56–0.99) | 0.62 (0.52–0.71) | -2.54 | 0.011* |
| GP22 | 0.19 (0.15–0.40) | 0.14 (0.10–0.19) | -3.695 | <0.001* |
| GP23 | 3.01 (2.34–14.02) | 2.52 (2.14–2.78) | -3.198 | 0.001* |
| GP24 | 1.78 (1.39–2.52) | 1.50 (1.23–1.79) | -2.8 | 0.005* |
| Derived traits | 65.06 (56.23–69.58) | 70.20 (67.83–72.25) | -4.346 | <0.001* |
| Stotal | 34.94 (30.42–43.78) | 29.81 (27.76–32.16) | -4.355 | <0.001* |
| S1 | 26.04 (22.98–28.23) | 24.39 (22.76–26.22) | -1.992 | 0.046* |
| S2 | 5.77 (4.81–19.84) | 4.92 (4.16–5.50) | -3.344 | 0.001* |
| F | 94.09 (91.44–95.71) | 94.96 (94.20–96.06) | -2.746 | 0.006* |
| FN | 95.72 (94.29–96.82) | 96.37 (95.06–96.98) | -1.651 | 0.099 |
| FS | 91.26 (87.48–93.59) | 92.95 (91.58–93.98) | -2.59 | 0.010* |
| B | 13.35 (11.45–14.30) | 12.78 (11.87–14.10) | -0.488 | 0.626 |
| BN | 11.03 (9.80–12.44) | 10.80 (9.73–11.90) | -1.011 | 0.312 |
| BS | 10.42 (8.99–12.33) | 10.23 (9.24–12.01) | -0.066 | 0.948 |
| FG0 | 8.55 (5.71–10.27) | 8.70 (7.29–10.73) | -1.313 | 0.189 |
| FG1 | 20.12 (17.67 - 22.51) | 23.50 (20.39–24.83) | -4.083 | <0.001* |
| FG2 | 23.66 (19.79–26.39) | 26.07 (23.73–27.36) | -3.123 | 0.002* |
| G0 | 10.65 (7.89–13.84) | 11.08 (9.55–13.50) | -1.34 | 0.18 |
| G1 | 24.70 (21.14–28.24) | 28.21 (26.18–29.67) | -3.876 | <0.001* |
| G2 | 27.93 (24.47–30.98) | 29.85 (27.66–32.41) | -2.56 | 0.010* |
| Gal-ratio | 0.13 (0.09–0.17) | 0.13 (0.11–0.16) | -0.093 | 0.926 |

*Statistically significant, *P* < 0.05. *P* values were calculated by the Mann‒Whitney U test. HIE, Hypoxic-ischemic encephalopathy; GP, glycan peak; N, neutral glycans; S, sialic acid; G, galactose; F, core fucose; B, bisecting GlcNAc.


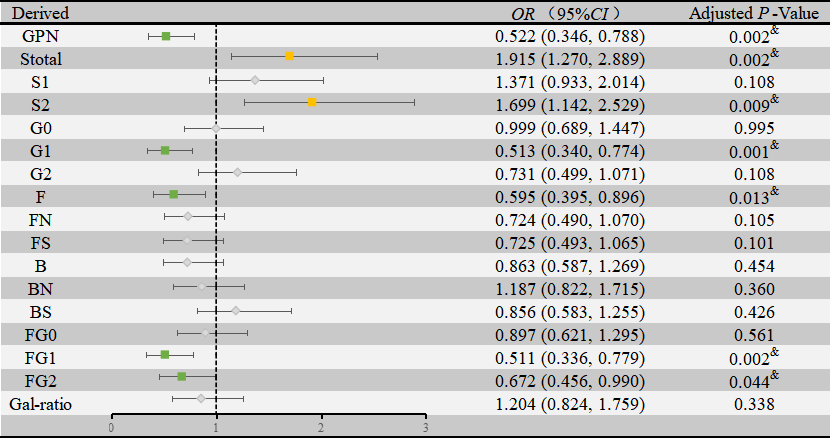


Figure S1. Associations of the derived traits and HIE as determined by multivariate logistic regression analyses.

Multivariate logistic regression analyses were performed after adjusting for the effects of fetal distress, low birth weight, and globulin, and *P*<0.05 was considered statistically significant using logistic regression analysis.

HIE, hypoxic-ischemic encephalopathy; N, neutral glycans; S, sialic acid; G, galactose; F, core fucose; B, bisecting GlcNAc; OR, odds ratio; CI, confidence interval.


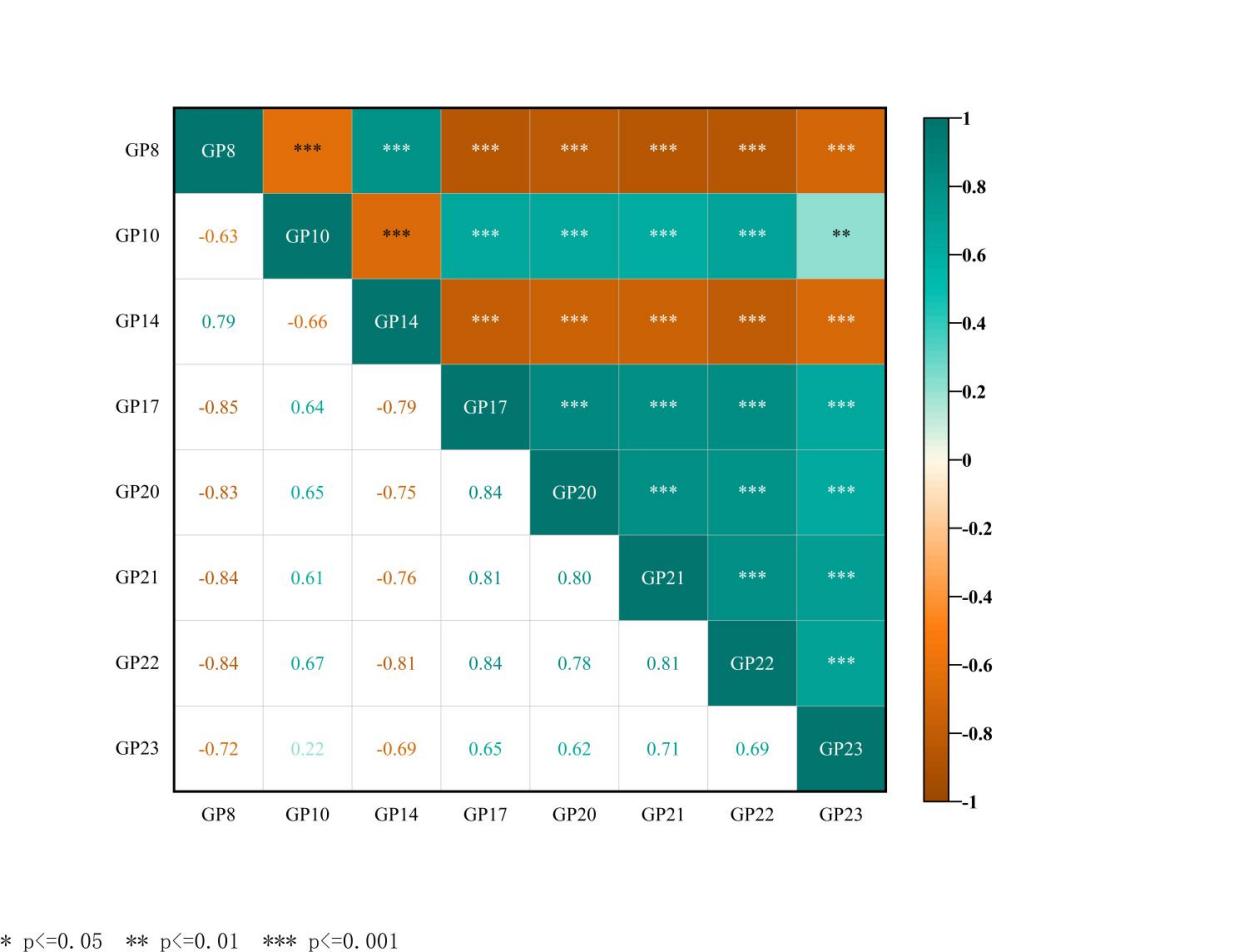


Figure S2. The correlation coefficient of independent glycans associated with HIE obtained by Spearman correlation analysis. Statistically significant, **P* < 0.05; ***P* < 0.01; ****P* < 0.001. *P* values were calculated by Spearman correlation analysis. The positive correlations are represented by green, while negative correlations are represented by brown. GP, glycan peak; HIE, Hypoxic-ischemic encephalopathy.

Table S3 Diagnostic models of HIE using IgG N-glycans and blood markers

| Variables | *β* | *SE* | Wald χ^2^ | *OR* (95%*CI*) | *P* |
| --- | --- | --- | --- | --- | --- |
| Model 1 |  |  |  |  |  |
| GP8 | -0.678 | 0.216 | 9.829 | 0.508 (0.332–0.776) | 0.002* |
| GP14 | -0.405 | 0.205 | 3.909 | 0.667 (0.447–0.996) | 0.048* |
| GP20 | 0.484 | 0.222 | 4.767 | 1.622 (1.051–2.504) | 0.029* |
| constant | 1.469 | 1.005 | 2.137 |  | 0.144 |
| Model 2 |  |  |  |  |  |
| fetal distress | 1.955 | 0.857 | 5.206 | 7.063 (1.317–37.868) | 0.023* |
| low birth weight infant | -0.977 | 0.491 | 3.952 | 0.377 (0.144–0.986) | 0.047* |
| globulin | -0.235 | 0.083 | 8.079 | 0.791 (0.672–0.930) | 0.004* |
| constant | 4.220 | 1.340 | 9.919 |  | 0.002* |
| Model 3 |  |  |  |  |  |
| GP8 | -0.759 | 0.207 | 13.381 | 0.468 (0.312–0.703) | <0.001* |
| fetal distress | 1.721 | 0.909 | 3.584 | 5.589 (0.941–33.186) | 0.058 |
| globulin | -0.231 | 0.089 | 6.762 | 0.794 (0.667–0.945) | 0.009* |
| constant | 5.321 | 1.499 | 12.597 |  | <0.001* |

*Statistically significant; *P* values were calculated by stepwise logistic regression.

*β*, regression coefficient; *CI*, confidence interval; HIE, Hypoxic-ischemic encephalopathy; *OR*, odds ratio; GP, glycan peak; *SE*, standard error.

Table S4 Evaluation of classification performance of three groups of models to distinguish neonatal encephalopathy from healthy control group

| Variables | Sensitivity | Specificity | Youden Index | AUC | 95%*CI* of AUC | |
| --- | --- | --- | --- | --- | --- | --- |
| Model 1 | 62.3% | 82.5% | 0.448 | 0.783 | 0.694 | 0.871 |
| Model 2 | 71.7% | 68.4% | 0.401 | 0.749 | 0.658 | 0.840 |
| Model 3 | 67.9% | 80.7% | 0.486 | 0.798 | 0.716 | 0.880 |

Model 1 consists of GP8, GP14 and GP20. Model 2 consists of fetal distress, low birth weight and globulin. Model 3 consists of GP8, fetal distress and globulin.

GP, glycan peak; AUC, area under the curve; *CI*, confidence interval; HIE, Hypoxic-ischemic encephalopathy; ROC, receiver operating characteristic curve.
